# Pathways for transforming biodiversity governance: An examination of the Global Biodiversity Framework’s Considerations

**DOI:** 10.1007/s13280-025-02215-8

**Published:** 2025-08-09

**Authors:** Alison Hutchinson, Anthony R. Zito, Philip J. K. McGowan

**Affiliations:** 1 School of Natural and Environmental Sciences, Newcastle University, Ridley Building 2, Newcastle-Upon-Tyne, NE2 4AA UK; 2 School of Geography, Politics and Sociology, Newcastle University, Ridley Building 2, Newcastle-Upon-Tyne, NE2 4AA UK

**Keywords:** Convention on Biological Diversity, Ecocentrism, Kunming-Montreal Global Biodiversity Framework, Rights of nature, Transformative implementation

## Abstract

With less than five years remaining to meet global commitments outlined in the Kunming-Montreal Global Biodiversity Framework (GBF)—to stem biodiversity loss—systemic and radical changes in biodiversity governance and management are needed. Addressing these challenges requires a fundamental and transformative shift in the way nature is valued across political, economic, and social spheres. We suggest that the GBF’s Considerations section provides a crucial foundation for this shift, but dedicated processes are needed to advance their more transformative elements. This paper untangles the norms and values underpinning the Considerations and discusses challenges and opportunities for integrating their most transformative aspects into biodiversity governance.

**Supplementary Information:**

The online version contains supplementary material available at 10.1007/s13280-025-02215-8.

## Introduction

### Background and context

Biodiversity is deteriorating at an unprecedented rate (IPBES 2019). It is well understood that species and ecosystems exist in a delicate balance, with losses producing rippling impacts on species’ population resilience, genetic diversity, and the well-being of living nature and humans alike. Anthropogenic pressures on climate, terrestrial, freshwater, and marine systems threaten approximately 47 000 known species (IUCN 2025) with extrapolations suggesting that over one million—largely undocumented—species are also at risk of extinction (notably, a conservative estimate) (IPBES 2019; Purvis 2019). In this growing context of biodiversity decline, it is increasingly recognised that business-as-usual policies and growth-based economic systems are no longer tenable solutions for planetary crises (Shin et al. 2019; Contestabile 2021; Turnhout et al. 2021; Friedman et al. 2022; Guterres 2022); transformative structural changes in political, technological, economic, and social realms are urgently needed (IPBES 2019; Fougères et al. 2022; Kok 2022).

This paper seeks to contribute to ongoing interdisciplinary and policy-facing conversations on the need for transformations across biodiversity governance by offering the first in-depth examination of the Considerations outlined in Section C of the Kunming-Montreal Global Biodiversity Framework (GBF). The Considerations are important for two reasons: first, they provide a focal point for how the Framework is to be ‘understood, acted upon, implemented, reported and evaluated’; and second, they introduce*—*for the first time in an international agreement—the recognition of the Rights of Mother Earth and the Rights of Nature^1^ (CBD 2022). Given their transformative potential, it is critical that the role of these Considerations in supporting the delivery of biodiversity Goals and Targets is examined with urgency.

We aim to initiate this process by opening a dialogue between policymakers, practitioners, academics, and civil society actors around the potential for these Considerations to reshape how biodiversity policy is conceptualised, implemented, and enacted. We begin by outlining the development of biodiversity governance interventions in the context of academic discussions on the structural processes, knowledge, and values shaping governance. We then describe the emergence of the Framework and trace the development of its Considerations, illustrating the opportunities and challenges for advancing transformative shifts in the operationalisation of the Framework. Finally, we propose pathways and practices—grounded in academic debate and evolving policy needs—intended to enable meaningful delivery of the GBF and drive system-wide change in biodiversity governance.

### Global policy context and the need for transformative governance

Global biodiversity policy is shaped by the UN Convention on Biological Diversity (CBD), which has near universal membership^2^ spanning 195 countries and the European Union. The CBD is governed by the Conference of the Parties (CoP) which meets biennially to set priorities and plans. This process is supported through annual meetings of three Subsidiary Bodies (SBs): the SB on Scientific, Technical and Technological Advice (SBSTTA), the SB on Implementation (SBI), and the newly formed SB for Traditional Knowledge, Innovations and Practices (SB8j) (we revisit these bodies later as mechanisms for shaping dialogue and shifting norms across the CBD).

The CBD has previously adopted two decadal strategies for biodiversity governance: the 2002–2010 Strategic Plan, and the 2011–2020 Strategic Plan—including the Aichi Biodiversity Targets. Both are largely understood to have failed in many aspects of implementation (CBD 2020). Scholars frequently cite a lack of political will and co-ordination, power asymmetries, and differing values for nature as key barriers to implementation progress (Morgera and Tsioumani 2010; Smallwood et al. 2022). These challenges reflect deeper tensions across biodiversity governance, which has traditionally been shaped by dominant (Western-based and industrial) norms and anthropocentric (human-centred) values that prioritise capitalist and utilitarian perspectives towards nature (Washington et al. 2021; Spash and Hache 2022). It has long been noted that achieving the CBD’s vision of ‘living in harmony with nature’ will require system-wide transitions to the normative foundations in biodiversity governance (see: CBD 14/9, Agenda item 17). If new ambitions for biodiversity governance are to succeed, historic and persistent tensions will need to be reduced.

### Transformative opportunities in the Global Biodiversity Framework

The GBF, agreed by parties to the CBD in 2022, is intended to mobilise collective efforts to tackle the biodiversity crisis (CBD 2022). The Framework establishes four long-term Goals for biodiversity restoration and governance to be achieved by 2050 and twenty-three Targets that describe actions to be implemented by 2030 (see Section G and H of the GBF). A notable innovation of the GBF—distinguishing it from previous Strategic Plans—is the inclusion of eighteen Considerations (see Table 1 and Section C of the GBF), which were referred to as ‘Principles’ and ‘Fundamental Premises’ during their development^3^ and are recognised as fundamental for the Framework’s implementation.

**Table 1:** Summary of the considerations listed in Section C of the GBF, organised into five core themes developed for the purpose of this analysis. See CBD (2022) for full descriptions. Icons correspond with those in Figs. 1 and 2

Theme	Summary of considerations	
1. Full and far-reaching participation and effort	A	*Contribution and rights of indigenous peoples and local communities* Respects the rights and knowledge of Indigenous People and local communities and encourages their full participation
	C	*Whole-of-government and whole-of-society approach* Encourages cooperation, participation, action, and implementation from all (government and society)
	E	*Collective effort towards the targets* Broad public support and collective effort at all levels
2. Recognition of diverse worldviews and knowledge systems	B	*Different value systems* The diverse value systems for nature and nature’s contributions to people are recognised
	L	*Science and innovation* Implementation is informed by scientific evidence, traditional knowledge, technology, and innovation
	O	*Formal and informal education* Transformative, innovative, transdisciplinary, and lifelong education is championed, recognising diverse worldviews and knowledge
	R	*Biodiversity and health* Implementation is guided by a One Health Approach and other holistic approaches, including equitable access and benefit sharing
3. Acknowledgement of rights, empowerment, and justice	G	*Human rights-based approach* A human rights-based approach, acknowledging the right to a clean, healthy, and sustainable environment is championed
	H	*Gender* Gender equality and the empowerment of women and girls is championed
	N	*Intergenerational equity* Principles of intergenerational equity are mindful of the needs of future generations and encourage participation from younger generations
4. Practicalities for sustainable economic development and capacity	D	*National circumstances, priorities, and capabilities* Contributions may be made according to national circumstances, priorities, and capabilities
	F	*Right to development* Responsible sustainable socioeconomic development when contributing to conservation and sustainable use
	K	*Principles of the Rio Declaration* Implementation should be guided by the principles of the Rio Declaration on Environment and Development
	P	*Access to financial resources* Adequate, predictable, and easily accessible financial resources
5. Consistency with internal and external governance and practices	I	*Fulfilment of the three objectives of the Convention and its Protocols and their balanced implementation* Implementation aligns with the CBD objectives
	J	*Consistency with international agreements or instruments* Implementation is cohesive and consistent with international obligations and agreements
	M	M. *Ecosystem approach* This approach, guided by a focus on entire ecosystems and biological communities, is endorsed by the CBD. Parties should use this framework when developing biodiversity strategies and action plans
	Q	*Cooperation and synergies* Implementation is coherent with CBD protocols and other biodiversity-related conventions and agreements (multi/inter/national, sub/regional, national)

These Considerations introduce ecocentric (nature-centred) concepts that challenge dominant anthropocentric perspectives by recognising the intrinsic value of all life and the legitimacy of diverse knowledge systems (Wu 2020). Although historically marginalised in biodiversity governance, ecocentric thinking is well developed among social, ecological, and conservation sciences (Washington et al. 2017; Piccolo et al. 2018; Taylor et al. 2020; Massarella et al. 2021). These principles also resonate in many Indigenous cosmologies, including the Quechuan *Sumak Kawsay* and concepts of *Buen Vivir*, the Bantu philosophy of *Ubuntu*, Japan’s *Satoyama*, and Māori concepts of *Kaitiakitanga* (Erazo Acosta 2022; McAllister et al. 2023; Pascual et al. 2023). Given the escalating challenges across more-than-human^4^ realms, a genuine engagement with the more boundary-pushing and ecocentric worldviews contained in the Considerations offers a valuable opportunity to bridge contrasting knowledge systems and values, to reshape dominant practices, and to support greater equity across the CBD policy landscape (Domínguez and Luoma 2020; Held 2023). Now that we have outlined the policy landscape, we next examine the Considerations before proposing approaches to support transformative shifts in the operationalisation of the Framework.

## The considerations in focus—opportunities and challenges to transform policy

To understand how the Considerations can motivate transformative change, we have grouped them by the following five core themes (also presented in Table 1):

1. Full and far-reaching participation and effort (Considerations A, C, E).
2. Recognition of diverse worldviews and knowledge systems (Considerations B, L, O, R).
3. Acknowledgement of rights, empowerment, and justice (Considerations G, H, N).
4. Practicalities for sustainable economic development and capacity (Considerations D, F, K, P).
5. Consistency with internal and external governance and approaches (Considerations I, J, M, Q).

While a number of Considerations focus on procedural and practical concerns (core themes 4 and 5), many support ecocentric concepts and challenge dominant biodiversity governance practices. Core themes 1, 2, and 3 emphasise the recognition of diverse values and worldviews, encourage greater participation, and promote holistic and ecocentric perspectives. These Considerations arguably have greater transformative potential than those addressing more operational issues such as calling for alignment with existing agreements and access to sufficient funding.

### Recognising diverging concepts underpinning the Considerations

The range of issues in the Considerations demonstrates the complexity of matters raised during the negotiation of the GBF. A focus on collective effort and participation (core theme 1) speaks to the need for enhancing the ability of less powerful groups to participate in the implementation of the Framework (for more on Global South inclusion, diverging worldviews, and power dynamics in the CBD, see: Morgera and Tsioumani 2010; Hall 2022; Smallwood et al. 2022; and Parks and Tsiomani, 2023). These Considerations additionally recognise the Rights of Indigenous Peoples and introduce ecocentric, non-market-based concepts of well-being which stand in stark contrast to traditional environmental conservation practices (Kopnina 2012; Goyes et al. 2021).

Core theme 2 focusses on integrating diverse approaches and further fosters engagement with contrasting values by juxtaposing traditional anthropocentric and economic-driven views of nature (e.g. natures’ goods and services) with more socially and ecologically just perspectives (e.g. natures’ gifts, the Rights of Nature, and One Health)—see also: Cariño and Ferrari 2021; Hall 2022; Kok et al. 2022; Parks and Tsioumani 2023. We return to the usefulness of these juxtapositions for supporting more inclusive and pluralistic policy development later in the text. Socially just transformations in biodiversity governance are further strengthened through the Considerations in core theme 3 which links human and intergenerational rights with concepts of environmental health and protection (albeit through a human-interest driven lens—see Padilla 2023).

In contrast, the Considerations focused on development practicalities (core theme 4) maintain links to dominant Western market-orientated conservation approaches where nature is viewed primarily in terms of the services and economies it provides (see Dancer 2021). While aligning the Framework with national development priorities reflects an awareness of the power and wealth disparities between Parties, development-orientated conservation approaches may produce perverse outcomes in practice, especially if focussed on short-term priorities and like-for-like compensation rather than preventing biodiversity loss (see also Duffy 2010; Spash 2015, 2020; Fajardo Del Castillo 2021; Zu Ermgassen et al. 2022).

Similar concerns also underscore the ‘mitigation hierarchy’ concept—a framework recognised by CBD Parties^5^ to quantify and scale up efforts for biodiversity impact management. While intended to guide the minimisation and avoidance of negative biodiversity outcomes, the approach raises additional challenges around compliance and the establishment of robust baselines and indicators—especially when biodiversity offsets are involved (see: Arlidge et al. 2018). Related tensions emerge in the final four Considerations on protocols and norms (core theme 5). Although prioritising multilateral cohesion with other biodiversity and climate agreements may enhance cooperation and coordination, a streamlining approach could inadvertently limit the Framework’s potential for transformative change if overshadowed by mainstream, anthropocentric-driven policies (Büscher and Fletcher 2019; Dancer 2021).

### Untangling contrasting ideologies in the Considerations

To bridge these conceptual differences, a critical reflection on the ideologies underpinning the Framework is needed. As a step towards this, we propose categorising the Considerations by two overarching contrasting positions: 1. Contrasting values: from *anthropocentrism* to *ecocentrism*, and 2. Foundations of knowledge: from *typical* (conventional), often positivistic approaches—that emphasise measurability, human-nature dualisms, and market-led reasoning, to more *transformational* concepts—rooted in Indigenous, eco-philosophical, and holistic worldviews.

**Fig. 1:**
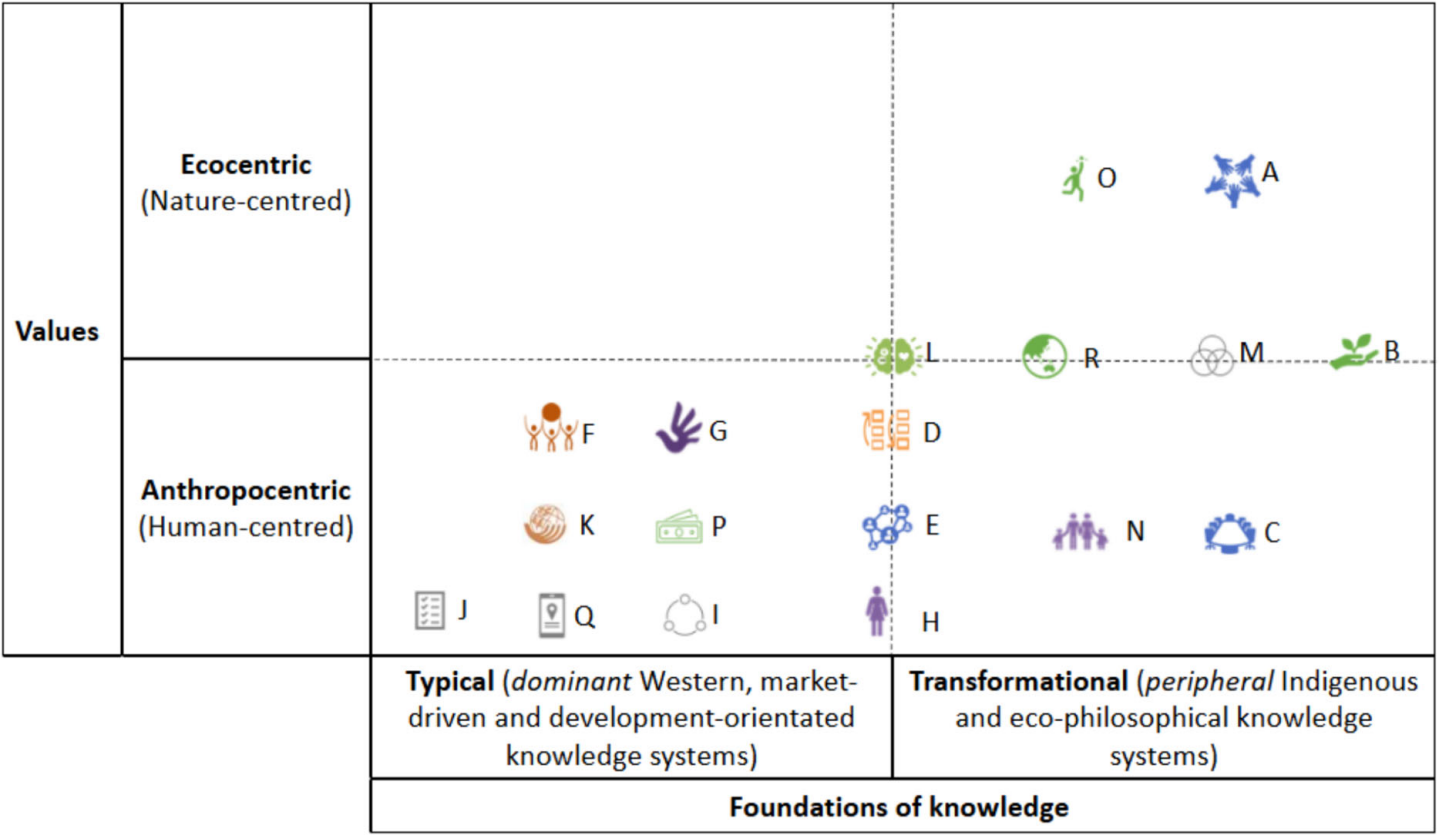
Illustration of the GBF’s Considerations showing the weighting of underpinning values and knowledge concepts. Note: while represented discretely, this grid is intended to convey gradients between values and knowledges. Western knowledge systems are not monolithic nor inherently anthropocentric, and Indigenous worldviews—while often offering alternatives to dominant Western environmental paradigms—are themselves diverse, spanning ecocentric and anthropocentric dimensions. This categorisation is not definitive but rather offers a starting point to examine the Framework’s underlying assumptions and encourage interdisciplinary and cross-cultural dialogue

The duality of approaches shown in Fig. 1 illustrates a diversity of values and knowledges in the Framework that may lead to tensions in implementation. The overall balance of perspectives (judged by number of Considerations) appears weighted towards anthropocentric values and typical knowledge systems (Fig. 1, lower left-hand quadrant). These concepts are largely incompatible with ecocentric values and transformational knowledge systems (Fig. 1, right-hand quadrant) (Kopnina et al. 2012; Piccolo et al. 2022). For example, concepts of sustainable development and use (Considerations F and K, as well as Sections D, Goal B, and Targets 9–13 in Section H of the GBF) will differ vastly depending on whether anthropocentric or ecocentric values inform the conceptual standpoints. An anthropocentric understanding of ‘sustainability’ promotes concepts of natural capital and ties business orientated and economic logics to nature conservation (Spash 2015; Fajardo Del Castillo 2021; Zu Ermgassen et al. 2022). Such framings are not necessarily sustainable or equitable for *all*; they fall short of broader ecocentric and holistic perspectives (Heydon 2019; Blaustein et al. 2020; McDonnell et al. 2020) and can fail to address broader notions of social, multi-species, planetary, and intergenerational justice,^6^ which will undoubtedly produce challenges for the effective and just implementation of the Framework.

Potential for transformative governance shifts can be seen through the introduction of ecocentric and holistic Considerations (Fig. 1, right-hand side). For example, recognising Indigenous Peoples’ rights (Consideration A) can expand mainstream concepts of well-being, which in many indigenous cosmologies (particularly Buen Vivir) are framed in terms of community and the interconnectedness between humans, other species, cultures,^7^ communities, ecosystems, and the natural world. As such, an Indigenous right for well-being extends a focus to all nature (beyond just humans or the environment). Similarly, One Health approaches (Consideration R) acknowledge the intrinsic value of all beings (beyond anthropocentric characterisations), bridging Western ecocentric thinking with Indigenous understandings (Washington et al. 2018; Pollowitz et al. 2024). These expanded notions of well-being introduce pathways to entwine social and ecological justice—so that *harmony with nature* becomes equally dependant on justice for nature and justice for people (see Washington et al. 2024), offering a more balanced approach to guide transformative shifts in how biodiversity governance is structured and performed.

### Why a balanced weighting in values and knowledges is important

The unequal balance of concepts, values, and knowledge foundations seen in the Considerations runs the risk that emerging science-policy outcomes may inadvertently endorse or prioritise the worldviews of cultural and political centres of power; with dominant Western knowledge production and rationality becoming the designated objective, irrespective of other heterogeneous ways of knowing (see: de Sousa Santos 2015; Goyes 2018). The development of the GBF’s Goals and Targets provides some indication of this. For example, the drive to ensure that 30% of terrestrial, inland water, and marine areas are effectively conserved by 2030 (Target 3) may encourage a ranking of ‘high-priority’ sites based on select attributes that fail to capture the broader, holistic needs of biodiversity conservation (concepts which are captured in other components of this Target). In practice, quantitative ambitions for area-based conservation frequently risk perverse outcomes—reinforcing inequalities, producing scientific expertise devoid of social context, and shaping directionality to serve the interests of powerful groups (see: White 2010; Barnes et al. 2018; Turnhout et al. 2020). It is critical, therefore, that diverse knowledge systems and values are given full attention to advance the non-quantitative elements of Target 3, such as equitable governance and the recognition of indigenous and traditional territorial rights, where applicable, so that decisions on how to conserve areas of biodiversity importance are not shaped solely by anthropocentric priorities.

In a similar vein, the dominance of mainstream scientific method is evident in Goal A of the GBF which calls for halting ‘human-induced extinction of *known* threatened species’ (emphasis added). While another clause in Goal A addresses the abundance of all wild native species, suggesting a more ecocentric approach, the emphasis on known threatened species^8^ reflects a reliance on data from tools like the IUCN Red List^9^ – the only globally available way for assessing species status. These quantitative resources, however, are widely recognised as taxonomically biased towards higher vertebrates^10^ and this narrow focus can fail to align with indigenous perspectives (Piccolo et al. 2022; Bocchi 2024). Despite being considerably resource constrained (see Juffe-Bignoli et al. 2016), efforts are underway to incorporate different knowledge systems and expand the knowledge base used in species assessments (see: IUCN 2022). Such efforts to improve inclusivity and representation may help mitigate what Turnhout and Purvis (2020, p. 675) describe as ‘the risk of mistaking what is easily counted for what counts, and overlooking what is not counted’.

## Pathways and practices to support transformative change

### Ensuring that the Considerations matter—generating normative change

The diverging ideologies and values highlighted in the above discussion indicate the potential for some of the more holistic (and demanding) elements in the Framework to be sidelined, especially considering the challenges and constraints facing many Parties (e.g. political will and agenda-setting, a lack of knowledge and implementation capacity, and unequal power and representation in international fora). While there is a general consensus on the urgent need to prevent further biodiversity losses, there remains a considerable implementation gap between academic research and real-world policy implementation—particularly between research that has practical consequences and that which is aspirational. This implementation gap is widened further by the persistent disconnect between ambitions set out in global biodiversity policy—what Parties acknowledge they must do to ‘live in harmony with nature’—and the practicalities of delivering and implementing those changes on the ground (see Knight et al. 2008; Koh et al. 2021; Friedman et al. 2022). Without coherent integration of the more transformative and ecocentric Considerations, continuation of business-as-usual approaches remains a strong possibility.

The challenge then for implementation is how to respond to contrasting and often conflicting perspectives to provide the best combined outcomes for nature, biodiversity, and people. If we are to instigate transformative change meaningfully, it will be necessary to recognise that the ecocentric and holistic perspectives articulated in the Considerations cannot be achieved when governance is conceptualised and enacted through short-term, anthropocentric, and market-driven frameworks that merely reconfigure the logic of the problem as the logic of the solution, without fundamentally addressing the root of the problem itself (Martin 2020; Büscher and Duffy 2023; Sène 2023). To drive normative change and integrate boundary-pushing concepts for ecological, multi-species, epistemic, and social justice, these perspectives must be supported to influence all stages of decision-making and practice.

### Implementation tools for transformative changes in biodiversity governance

**Fig. 2:**
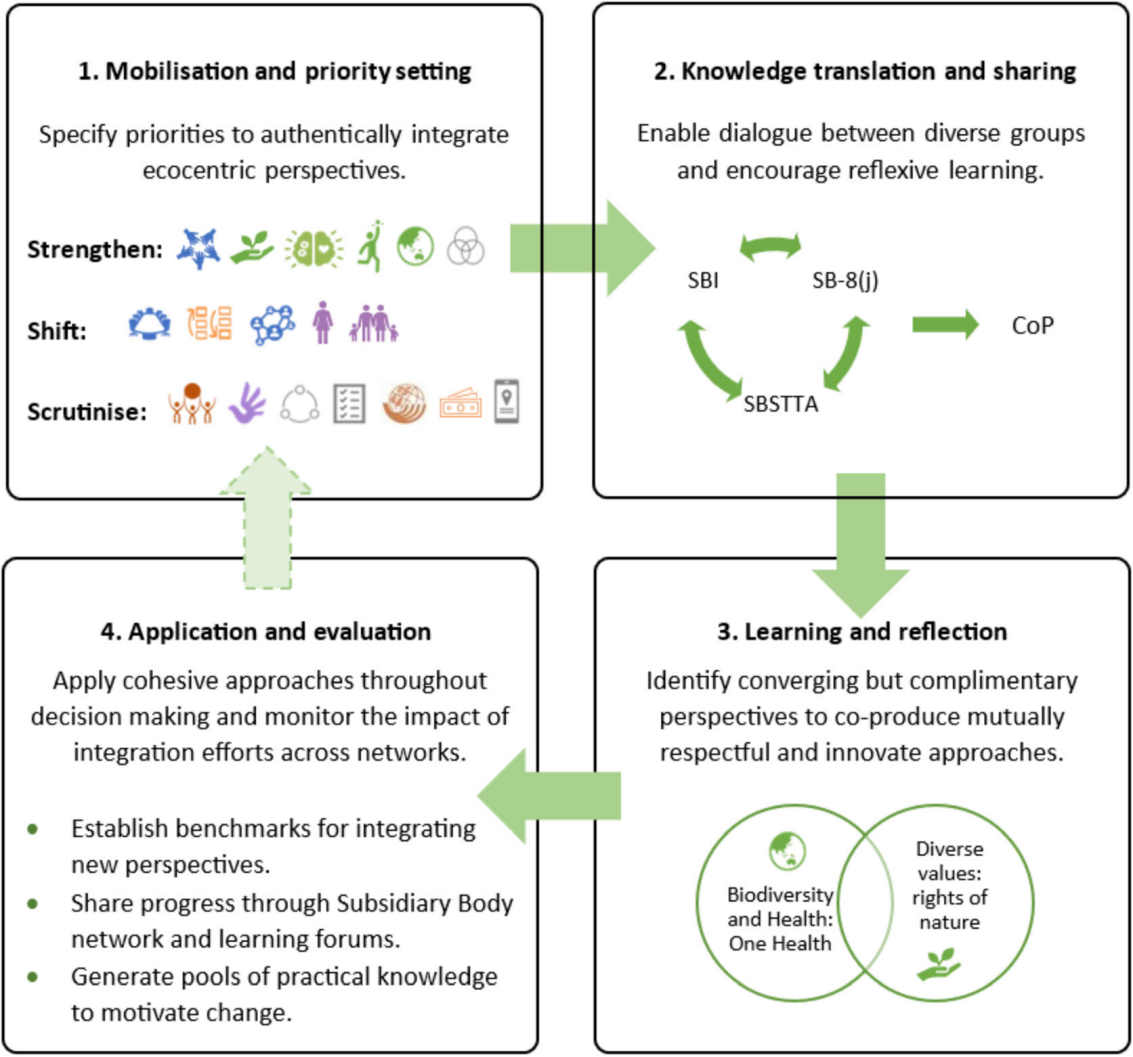
Suggested pathways to encourage a normative expansion process and the incorporation of transformative ecocentric approaches and perspectives both within the CBD regime and everyday political practices. Processes described here have been adapted from Tengö et al. (2017) and Jordan and Lenschow (2010) whose combined approaches demonstrate how facilitating dialogue and exchange can aid the development of effective knowledge sharing and learning frameworks

Given that the CBD’s governance has evolved through principles of soft law and iterative negotiation (Harrop and Pritchard 2011), and if each CBD CoP is seen as an individual step in this ongoing process, the integration of transformative and ecocentric perspectives would benefit from explicit highlighting across all levels of CBD activity—including CoPs, SBs, and expert and advisory groups—as well as through broader ‘whole-of-society’ (i.e. Consideration (c) networks). While opportunities for normative and ecocentric shifts will evolve in response to the needs of CBD Parties and pressures on the natural world, we suggest that this process may be expedited by promoting adaptive learning frameworks, supported by inclusive governance and engagement. One way to support the translation, negotiation, and synthesis of diverse perspectives and promote a shift in norms and values across CBD, national, and local levels would be through the design of ratcheting mechanisms that give space for learning and encourage the incorporation of the Considerations into policy and practice—for example, through successive CoP cycles and the development of National Biodiversity Strategies and Action Plans (NBSAPs). Similar practices have been effectively developed in climate governance. Figure 2 illustrates how this process may unfold in practice.

This four-stage approach represents a process of learning and exchange over time. To begin with mobilisation and priority setting (Stage 1, Fig. 2), we suggest that attention could follow three paths: 1. strengthening Considerations that encompass transformative and ecocentric perspectives (top-right box, Fig. 1), 2. shifting transformative—yet human-centred—Considerations (centre-line and lower-right box, Fig. 1) to engage with eco-philosophical concepts, and 3. scrutinising anthropocentric and traditionally aligned Considerations (lower left box, Fig. 1) to motivate a transition to transformative and ecocentric approaches.

The translation and sharing of knowledge and values into appropriate formats (Stage 2, Fig. 2), would be strengthened by embedding community and Indigenous voices, as well as fostering pluralist dialogue among state, substate, non-state, and other interested parties—both in formal meetings and informal dialogues and networking of the CBD (sensu Domorenok and Zito 2021). This may, for example, draw on the establishment of SB8j,^11^ a new CBD subsidiary body, that is intended to provide a useful platform to bridge diverging knowledge and value systems. Although still under development and subject to consideration at CoP-17, SB8j is already included in the Convention’s workplan on indigenous peoples and local communities.^12^ As its proposed modus operandi will be considered at COP-17 (see footnote 11), there is an urgent need to agree on mechanisms that support meaningful engagement with diverse worldviews.

While recognising and supporting ecocentric perspectives in the formal CBD meetings will be important, equally critical will be engagement with both formal and informal multilateral networks that enable ecocentric thinkers across cultures to engage with other ecocentric viewpoints, as well as with practitioners who adhere to anthropocentric perspectives. The recently established CBD regional and subregional ‘Technical and Scientific Support Centres’ offer one such opportunity to facilitate discussion between Parties. More broadly, spaces for dialogue may be cultivated through the CBD’s caucuses and observer groups, including the academic and research caucus (A&R Group), the indigenous peoples’ caucus (IIFB), and Indigenous Peoples Observer organisations, as well as across wider multilateral, governmental, civil society, and community-based institutions. These varied platforms provide opportunities to strengthen the ability of less powerful and marginalised groups to shape and inform decision-making (see Zafra-Calvo, 2020), while also promoting avenues for consensus building, knowledge exchange, and training across diverse perspectives (Victor 1998; Mewes and Unger 2021) to collectively establish a ‘learning by doing’ approach for integrating the Considerations.

To enable the co-production of common understandings between contrasting perspectives (e.g. between anthropocentric norms and transformative ecocentrism), the above-mentioned networks will need to be developed and strengthened over time (Stage 3, Fig. 2). Supporting these networks can enable the co-production of shared perspectives to catalyse new pathways for transformative policy and practice. For example, Consideration B (diverse values and Rights of Nature) and Consideration R (One Health) offer complementary pathways to integrate ecocentric values; with the former rooted in Indigenous knowledge systems, and the latter in Western eco-philosophical tradition. The exchange of ideas and negotiation of converging and complementary perspectives can be supported through the development of capacity building and knowledge exchange across the CBD’s institutional architecture (at CoP, SB meetings, across expert and advisory groups, and other events) and within the broader policy landscape (including platforms such as the Intergovernmental Science-Policy Platform on Biodiversity and Ecosystem Services—IPBES, national institutions, governing bodies, non-governmental organisations, local and community groups, etc.).

Incorporating holistic and ecocentric values into biodiversity governance and national implementation will require conscious reinforcement and repeat evaluation of these values both within and beyond the GBF (Stage 4, Fig. 2). Reporting and evaluation mechanisms that set benchmarks for integrating transformative ecocentric perspectives, and tools that allow Parties to share their measures, experiences and lessons learned, will be essential for tracking progress. It is important, therefore, that Parties have agreed that progress in addressing the Considerations be included in reporting on GBF implementation, by both Parties themselves and non-State actors.^13^

Learning forums, networks, and other platforms can help the CBD community exchange experiences, integrate ecocentric values, and reconcile some of the tensions inherent in current efforts. These forums will also provide practical knowledge on successful local initiatives. By comparing progress, Parties may be inspired to adopt increasingly ambitious approaches for future CoPs. Negotiators may benefit from these ‘learning forums’ where shared experiences and interaction can help inform perspectives and encourage the integration of new transformative ideas (a similar learning process has facilitated breakthroughs in climate negotiations, see for example, Rietig 2019).

## Final remarks

The political negotiations underpinning the GBF reflect the prevalent values held towards nature. A track record of poorly performing and ineffective CBD Strategic Plans, and calls for transformative shifts in the values and paradigms underpinning biodiversity governance (IPBES 2019) highlight how business-as-usual responses are no longer an option if we are to seriously address species loss and biodiversity decline. Issues around the marginalisation of people and nature and the combined issues of human, gender, intergenerational, and ecological injustice have been under-established in environmental policy discourse (Francis 2020). While we acknowledge that political will and constraints in both time and capacity will influence the implementation of the GBF, a fundamental shift in approaches will be essential to meet the Framework’s shared vision of ‘living in harmony with nature’.

To support the expansion of effective, justice-informed, and holistic implementation approaches, this paper has emphasised the importance of the Considerations, teased out juxtapositions and tensions between them, and suggested pathways for more holistic implementation efforts. We maintain that the ecocentric approaches introduced in the Considerations provide areas to foster growth and transformative change in biodiversity governance, supporting arguments made by Washington et al. (2024). These approaches extend legal, procedural, and policy dimensions to recognise and uphold the rights of nature and biodiversity, while placing social and ecological justice at the core of future efforts. This provides the opportunity for Parties, practitioners, academics, local community, and interest groups to reflect on how their knowledges and values may influence GBF implementation, and advocate for the authentic integration of holistic and ecocentric practices that have been introduced in the GBF’s Considerations.

## Supplementary Information

Below is the link to the electronic supplementary material.

Supplementary file1 (DOCX 34 KB)

## Data Availability

We do not analyse or generate any datasets, all data supporting the findings of this study are available within the paper and its Supplementary Materials.

## Footnotes

1 Notably, while the Framework states that upholding these rights is essential for the Framework’s success, the language is nuanced, and such concepts apply only—’for those countries that recognize them’ (CBD 2022, p5).

2 Exceptions here include the USA and the Holy See.

3 See our Supplementary Information for the final adopted text, and the recommendations adopted by the Convention’s two subsidiary bodies, in advance of COP-16, with links to all relevant documents.

4 The concept of ‘more-than-human’ focusses attention on the interconnected relationships between all beings and species—humans, nature, and the environment (O’Gorman and Gaynor, 2020). Policy and governance circles are gradually recognising this language; in 2021 the CBD shared ‘Actions by Environmental Peacebuilders’ which discussed a more-than-human approach for improving biodiversity protection. The IPBES Values Assessment (2022, see Chapter 2—Conceptualizing the diverse values of nature and their contributions to people) additionally emphasised that relational and intrinsic values capturing more-than-human relationships need prioritisation in political decision making. The first UK-based interspecies council, established by DEFRA Futures Policy Lab (UK), has additionally recognised more-than-human natures (see: ‘Water Post 2043—Exploring divergent futures’).

5 See CBD Decision 14/3 ‘Mainstreaming of biodiversity in the energy, mining, infrastructure, manufacturing and processing sectors (CBD 2018).

6 For more on expansive concepts of justice see: Lerner and Berg (2017); Washington et al. (2018); Celermajer et al. (2020); Washington and Maloney (2020); Biermann (2021).

7 The importance of conserving animal culture and social knowledge is increasingly recognised as a means to improve conservation outcomes and make global biodiversity conservation more effective (Brakes et al. 2021; Lappe-Osthege and Duffy 2024). For example, the UN Convention on Migratory Species is the first global convention to acknowledge non-human (animal) cultural diversity and recommend that ‘[animal] cultural repositories and capacities should be integrated into the development of IUCN, CMS, and other conservation strategies’ (UNEP-CMS2018a, b, p2.).

8 See McGowan et al. (2024) for a discussion of these and other terms.

9 The IUCN Red List has been in existence since the 1960s and its current extinction risk categories and criteria have been used since 1994 (Mace et al. 2008).

10 Only 2 per cent of invertebrates, 8 per cent of fungi and protists, and 18 per cent of plants have been evaluated on the IUCN Red List, compared to 84 per cent of described vertebrates (accurate as of April 2025—see IUCN Red List version 2025–1 Table 1a).

11 SB8j is a new CBD subsidiary body to ensure the full and effective participation of Indigenous Peoples and local communities, see CBD Decision 16/5 (2024) ‘Institutional arrangements for the full and effective participation of indigenous peoples and local communities in the work undertaken under the Convention on Biological Diversity.

12 See CBD Decision 16/4 (2024) ‘Programme of work on Article 8(j) and other provisions of the Convention on Biological Diversity related to indigenous peoples and local communities to 2030’.

13 See CBD Decision 16/32 (2025) ‘Mechanisms for planning, monitoring, reporting and review, including the global review of collective progress in the implementation of the Kunming-Montreal Global Biodiversity Framework to be conducted at the seventeenth and nineteenth meetings of the Conference of the Parties’.
