# Supplementary material for: Pathways for transforming biodiversity governance: An examination of the Global Biodiversity Framework’s Considerations
Source: Ambio. 2025 Aug 9;55(1):56–67. doi: 10.1007/s13280-025-02215-8 (PMC12673004; doi:10.1007/s13280-025-02215-8)
Supplement: Supplementary file 1 — Supplementary file1 (DOCX 34 KB) [file 13280_2025_2215_MOESM1_ESM.pdf]

SUPPLEMENTARY MATERIAL:

This supplementary information has not been peer reviewed.

TITLE:

Pathways for transforming biodiversity governance: An examination of the Global Biodiversity Framework's 'Considerations'

AUTHORS:

Alison Hutchinson (Newcastle University, alison.hutchinson@newcastle.ac.uk), Anthony Zito, Philip JK McGowan.

JOURNAL:

Ambio

Supplementary Material

Development of Considerations in the Kunming-Montréal Global Biodiversity Framework

The need for a separate section that would become *Section C: Considerations* was first discussed in the 3rd meeting of the Open-ended Working Group on the post-2020 Global Biodiversity Framework (OEWG) (see CBD 2022a). At that time, the heading of the proposed section was suggested as *Principles and Approaches [Guidance]* for the implementation of the framework, indicating the fundamental importance of the content of this section, as it should underpin how the Framework is operationalised. Once issues deemed by various Parties to be fundamental to the Framework were brought into a single section, focus on that section increased (see supplementary information) and an informal working group meeting between the 4th and 5th meetings of the OEWG produced draft text that identified 17 issues that were classified as cross-cutting issues, principles or underlining premises (CBD 2022b). The use of the word 'principles' was discussed during this meeting along with potential legal implications of its use. Also deliberated upon was the way that 'common but differentiated responsibilities (CBDR)' was reflected in various multilateral agreements: CBDR is Principle 7 of the Rio Earth Declaration (UN 1993) and has underlying concepts of fairness and equity, stating that 'States shall cooperate in a spirit of global partnership to conserve, protect and restore the health and integrity of the Earth's ecosystem'.

ZERO DRAFT OF THE POST-2020 GLOBAL BIODIVERSITY FRAMEWORK
Open-Ended Working Group 2 Rome 24-29 February 2020
CBD/WG2020/2/3 6 January 2020
https://www.cbd.int/doc/c/da8c/9e95/9e9db02aaf68c018c758ff14/wg2020-02-03-en.pdf
Section B: Purpose contains three paragraphs, each starting with the words: "The Framework aims to …" or "The Framework will …"

UPDATED ZERO DRAFT OF THE POST-2020 GLOBAL BIODIVERSITY FRAMEWORK
CBD/POST2020/PREP/2/1 17 August 2020
https://www.cbd.int/doc/c/3064/749a/0f65ac7f9def86707f4eaefa/post2020-prep-02-01-en.pdf
Unchanged from the Zero Draft

FIRST DRAFT OF THE POST-2020 GLOBAL BIODIVERSITY FRAMEWORK
Open-Ended Working Group 3 Online, 23 August – 3 September 2021
CBD/WG2020/3/3 5 July 2021
https://www.cbd.int/doc/c/914a/eca3/24ad42235033f031badf61b1/wg2020-03-03-en.pdf
Section B: Purpose contains two paragraphs, each starting with the words: "The Framework aims to …"

The third paragraph from the Updated Zero Draft was put in Section C: Relationship with 2030 Agenda for Sustainable Development

REPORT OF THE OPEN-ENDED WORKING GROUP ON THE POST-2020 GLOBAL BIODIVERSITY FRAMEWORK ON ITS THIRD MEETING
Open-Ended Working Group 3 (part II) Geneva, Switzerland, 14–29 March 2022
CBD/WG2020/3/7
https://www.cbd.int/doc/c/50c9/a685/3844e4030802e9325bc5e0b4/wg2020-03-07-en.pdf
The report of the meeting included an appendix containing a proposal from the Co-Chairs for a new section (B.bis) of the post-2020 global biodiversity framework, presented in document CBD/WG2020/3/6, together with submissions from delegates for changes to or additional elements to this new section. The elements were included as submitted and were not discussed in contact

group 1. Some Parties and observers expressed the view that Section B.bis should not result in removal of important principles and standards (such as rights-based approaches, rights of indigenous peoples and local communities, and gender and youth) from the goals, targets and other sections of the framework, as appropriate.

Section B.bis began [alternative wording suggested in []
[title:] B.bis Principles and Approaches [Guidance] for the implementation of the framework

[chapeau:] The following principles and approaches were [guidance was] used in the development of the global biodiversity framework and should guide and underpin its implementation:

REPORT OF THE OPEN-ENDED WORKING GROUP ON THE POST-2020 GLOBAL BIODIVERSITY FRAMEWORK ON ITS FOURTH MEETING
Open-Ended Working Group 4 Nairobi, 21–26 June 2022
CBD/WG2020/4/4 21 June 2022
https://www.cbd.int/doc/c/3303/d892/4fd11c27963bd3f826a961e1/wg2020-04-04-en.pdf

Section B.bis in a separate Section, indicating that there should be some notion of principles or underpinning perspectives to the implement of the Framework. The potential titles and introduction (chapeau) were proposed as follows.

[Section B Bis [Principles and] [Approaches] [Guidance] for the implementation of the framework
7. The following [principles and] [approaches] [guidance] should underpin the implementation of the framework:

OUTCOMES OF THE WORK OF THE INFORMAL GROUP ON THE POST-2020 GLOBAL BIODIVERSITY FRAMEWORK
Informal Group on the Post-2020 Global Biodiversity Framework, Montreal, Canada, 26-30 September 2022
CBD/WG2020/5/2 https://www.cbd.int/doc/c/dfeb/e742/b936c09eae9dd558c1310b5b/wg2020-05-02-en.pdf

Considered Section B.bis in detail and what its purpose may be, viz: *The Group agreed that this section could provide very important context and foundational elements for the entire framework, including on how it should be implemented. In this context some suggested that the formulation of the section should provide an unambiguous articulation of the specific purpose and intended content of this section.*

There was discussion over the title of the section, perhaps referring to ‘principles’ for the implementation of the Framework. There was, however, concern about the potential legal nature of ‘principles; and so ‘premises’ or ‘guidance’ were suggested. There was discussion about the reflection of “common but differentiated responsibilities” in various multinational agreements and in the context of the Convention. A list of 17 issues were identified as such cross-cutting issues, principles or underlining premises.

As a result the Co-chairs of the Open-ended Working Group developed text for 13 ‘fundamental premises for the implementation of the framework’, which formed the basis of discussion at OEWG 5.

RECOMMENDATION ADOPTED BY THE WORKING GROUP ON THE POST-2020 GLOBAL BIODIVERSITY FRAMEWORK

Open-Ended Working Group 5 Montreal 3-5 December 2022
CBD/WG2020/REC/5/1 5 December 2022 https://www.cbd.int/doc/recommendations/wg2020-05/wg2020-05-rec-01-en.pdf

Section Bbis was only partially discussed by the contact group at OEWG 5 and much of the text remained in square brackets (ie not agreed by consensus). Importantly, it states that the Framework is to be acted upon, implemented, reported and evaluated, consistent with these 'principles/ premises/guidelines and approaches'. In other words, they should inform every stage of action concerning the KMGBF.

Their recommendation for this section began:

Section B bis. [The fundamental [premises]/[principles]/[guidelines and approaches] for the implementation of the framework*

[8. The implementation of the framework, including its Goals and Targets, is underpinned by fundamental [premises]/[principles]/[guidelines and approaches] that are key for its success [and are to be considered at all stages[, including planning, monitoring, reporting, and review]. [The comprehensive implementation of the framework includes consideration of these [premises]/[principles]/[guidelines and approaches] at all stages. In that regard, the Goals and Targets are to be understood, acted upon, implemented, reported and evaluated, consistent with the followings]]:]

There then follows 13 suggested issues, and a further eight drawn from the report of OEWG4.

DECISION ADOPTED BY THE CONFERENCE OF THE PARTIES TO THE CONVENTION ON BIOLOGICAL DIVERSITY 15/4. Kunming-Montreal Global Biodiversity Framework
CoP 15 Part II Montreal, Canada, 7-19 December 2022
CBD/COP/DEC/15/4 19 December 2022 https://www.cbd.int/doc/decisions/cop-15/cop-15-dec-04-en.pdf

Section C. Considerations for the implementation of the Kunming-Montreal Global Biodiversity Framework

7. The Kunming-Montreal Global Biodiversity Framework, including its Vision, Mission, Goals and Targets, is to be understood, acted upon, implemented, reported and evaluated, consistent with the following:

Contribution and rights of indigenous peoples and local communities

(a) The Framework acknowledges the important roles and contributions of indigenous peoples and local communities as custodians of biodiversity and as partners in its conservation, restoration and sustainable use. The Framework's implementation must ensure that the rights, knowledge, including traditional knowledge associated with biodiversity, innovations, worldviews, values and practices of indigenous peoples and local communities are respected, and documented and preserved with their free, prior and informed consent,[1] including through their full and effective participation in decision-making, in accordance with relevant national legislation, international

---

[1]In this framework, free, prior and informed consent refers to the tripartite terminology of "prior and informed consent" or "free, prior and informed consent" or "approval and involvement".

instruments, including the United Nations Declaration on the Rights of Indigenous Peoples,[2] and human rights law. In this regard, nothing in this framework may be construed as diminishing or extinguishing the rights that indigenous peoples currently have or may acquire in the future;

Different value systems

(b) Nature embodies different concepts for different people, including biodiversity, ecosystems, Mother Earth, and systems of life. Nature's contributions to people also embody different concepts, such as ecosystem goods and services and nature's gifts. Both nature and nature's contributions to people are vital for human existence and good quality of life, including human well-being, living in harmony with nature, and living well in balance and harmony with Mother Earth. The Framework recognizes and considers these diverse value systems and concepts, including, for those countries that recognize them, rights of nature and rights of Mother Earth, as being an integral part of its successful implementation;

Whole-of-government and whole-of-society approach

(c) This is a framework for all - for the whole of government and the whole of society. Its success requires political will and recognition at the highest level of government and relies on action and cooperation by all levels of government and by all actors of society;

National circumstances, priorities and capabilities

(d) The goals and targets of the Framework are global in nature. Each Party would contribute to attaining the goals and targets of the Framework in accordance with national circumstances, priorities and capabilities;

Collective effort towards the targets

(e) The Parties will catalyse implementation of the Framework through mobilization of broad public support at all levels;

Right to development

(f) Recognizing the 1986 United Nations Declaration on the Right to Development,[3] the Framework enables responsible and sustainable socioeconomic development that, at the same time, contributes to the conservation and sustainable use of biodiversity;

Human rights-based approach

(g) The implementation of the Framework should follow a human rights-based approach, respecting, protecting, promoting and fulfilling human rights. The Framework acknowledges the human right to a clean, healthy and sustainable environment;[4]

Gender

(h) Successful implementation of the Framework will depend on ensuring gender equality and empowerment of women and girls, and on reducing inequalities;

Fulfilment of the three objectives of the Convention and its Protocols and their balanced implementation

(i) The goals and targets of the Framework are integrated and are intended to contribute in a balanced manner to the three objectives of the Convention on Biological Diversity. The Framework is to be implemented in accordance with these objectives, with the provisions of the Convention on

---

[2] A/RES/61/295.

[3] A/RES/41/128.

[4] UN General Assembly Resolution 76/300 of 28 July 2022.

Biological Diversity, and with the Cartagena Protocol on Biosafety and the Nagoya Protocol on Access and Benefit-sharing, as applicable;

Consistency with international agreements or instruments

(j) The Framework needs to be implemented in accordance with relevant international obligations. Nothing in this Framework should be interpreted as agreement to modify the rights and obligations of a Party under the Convention or any other international agreement;

Principles of the Rio Declaration

(k) The Framework recognizes that reversing the loss of biological diversity, for the benefit of all living beings, is a common concern of humankind. Its implementation should be guided by the principles of the Rio Declaration on Environment and Development;[5]

Science and innovation

(l) The implementation of the Framework should be based on scientific evidence and traditional knowledge and practices, recognizing the role of science, technology and innovation;

Ecosystem approach

(m) This Framework is to be implemented based on the ecosystem approach of the Convention;[6]

Intergenerational equity

(n) The implementation of the Framework should be guided by the principle of intergenerational equity which aims to meet the needs of the present without compromising the ability of future generations to meet their own needs and to ensure meaningful participation of younger generations in decision-making processes at all levels;

Formal and informal education

(o) Implementation of the Framework requires transformative, innovative and transdisciplinary education, formal and informal, at all levels, including science-policy interface studies and lifelong learning processes, recognizing diverse world views, values and knowledge systems of indigenous peoples and local communities;

Access to financial resources

(p) The full implementation of the Framework requires adequate, predictable and easily accessible financial resources;

Cooperation and synergies

(q) Enhanced collaboration, cooperation and synergies between the Convention on Biological Diversity and its Protocols, other biodiversity-related conventions, other relevant multilateral agreements and international organizations and processes, in line with their respective mandates, including at the global, regional, subregional and national levels, would contribute to and promote the implementation of the Framework in a more efficient and effective manner;

Biodiversity and health

(r) The Framework acknowledges the interlinkages between biodiversity and health and the three objectives of the Convention. The Framework is to be implemented with consideration of the One Health Approach, among other holistic approaches that are based on science, mobilize multiple sectors, disciplines and communities to work together, and aim to sustainably balance and

---

[5] Rio Declaration on Environment and Development (A/CONF.151/26/Rev.l (vol.I)), United Nations publication, Sales No. E.93.1.8.

[6] Decision V/6.

optimize the health of people, animals, plants and ecosystems, recognizing the need for equitable access to tools and technologies including medicines, vaccines and other health products related to biodiversity, while highlighting the urgent need to reduce pressures on biodiversity and decrease environmental degradation to reduce risks to health, and, as appropriate, develop practical access and benefit-sharing arrangements.

POST COP15

RECOMMENDATIONS ADOPTED BY THE SUBSIDIARY BODY ON SCIENTIFIC, TECHNICAL AND TECHNOLOGICAL ADVICE
Subsidiary Body on Scientific, Technical And Technological Advice 25 Nairobi 15–19 October 2023
CBD/SBSTTA/25/13 19 October 2023
https://www.cbd.int/doc/c/698b/dca4/dadb1b93ace9acae10f1bb04/sbstta-25-13-en.pdf

I Recommendations adopted by the Subsidiary Body on Scientific, Technical and Technological Advice
25/1. Monitoring framework for the Kunming-Montreal Global Biodiversity Framework

The Subsidiary Body on Scientific, Technical and Technological Advice …
9. *Encourages* the Expert Group to take **section C** of the Framework into consideration when addressing the gaps in the monitoring framework in preparation for the twenty-sixth meeting of the Subsidiary Body on Scientific, Technical and Technological Advice;

25/2. Scientific, technical and technological inputs that should inform the global review of collective progress in the implementation of the Kunming-Montreal Global Biodiversity Framework

The Subsidiary Body on Scientific, Technical and Technological Advice ….
1 *Decides* that the global review of collective progress in the implementation of the Kunming-Montreal Global Biodiversity Framework will be a process comprising several elements, including a global report focussed primarily on assessing progress in the implementation of the Framework and containing the following elements in its structure:
….

c A review of collective progress in the implementation of the Framework, including a target-by-target assessment of progress towards the 23 targets, the 2030 Mission and other elements of the Framework, including **sections C**, I, J and K;

RECOMMENDATIONS ADOPTED BY THE SUBSIDIARY BODY ON SCIENTIFIC, TECHNICAL AND TECHNOLOGICAL ADVICE
Subsidiary Body on Scientific, Technical And Technological Advice 26 Nairobi 13–18 May 2024

Recommendation adopted by the Subsidiary Body on Scientific, Technical and Technological Advice on 18 May 2024 18 May 2024 https://www.cbd.int/doc/recommendations/sbstta-26/sbstta-26-rec-02-en.pdf

26/2. Scientific and technical needs to support the implementation of the Kunming-Montreal Global Biodiversity Framework

The Subsidiary Body on Scientific, Technical and Technological Advice …

2. Concludes that most of the guidance that has been developed under the Convention on Biological Diversity is relevant and that there is a wealth of tools and guidance developed through other processes that also support the implementation of the Framework and the potential to work with them to mainstream the **considerations** for the implementation of the Framework[7] into their activities, and that access to guidance, adequate financial resources, capacity-building and development, and technical and scientific cooperation are needed by Parties for the implementation of the tools and guidance;

6. Recommends that, at its sixteenth meeting, the Conference of the Parties consider adopting a decision along the following lines, noting that complementary recommendations may be elaborated by the Subsidiary Body on Implementation:

The Conference of the Parties …

Welcoming the establishment of the regional and subregional technical and scientific cooperation support centres and the global knowledge support service for biodiversity,

1. *Recognizes:*
(d) That there is an opportunity to mainstream the Framework into the work undertaken under other relevant intergovernmental agreements and processes and by international organizations, the private sector and other stakeholders, including indigenous peoples and local communities, women and youth, and into capacity-building and development initiatives, with a view to integrating the **considerations** for the implementation of the Framework6[8] into tools and guidance developed through such processes and initiatives, so that they support biodiversity-inclusive actions and outcomes;

4. Requests the Secretariat, subject to the availability of resources: …
(b) To continue to facilitate the development of further tools [and exchange of good practices][and guidance and updating of existing guidance,]where needed, to address gaps identified [by Parties] [in annex III to document CBD/SBSTTA/26/2, section IV.B of and the annex to document CBD/SBSTTA/26/3 and document CBD/SBSTTA/26/INF/15], in cooperation with relevant multilateral environmental agreements, [other competent intergovernmental organizations,] [the regional and subregional technical and scientific cooperation support centres and the global knowledge support service for biodiversity], relevant processes and organizations, [avoiding duplication of tools and guidance,] and in consultation with Parties, other Governments, indigenous peoples and local communities, women and youth organizations and relevant stakeholders, and, where appropriate, [and in line with their respective mandates,] to work with such relevant processes and organizations to integrate the **considerations** for the implementation of the Framework into tools and guidance being developed under those processes and by those organizations in order to support biodiversity-inclusive actions and outcomes;

RECOMMENDATIONS ADOPTED BY THE SUBSIDIARY BODY ON IMPLEMENTATION
Subsidiary Body on Implementation 4 Nairobi 21–29 May 2024
CBD/SBI/REC/4/1 29 May 2024

Recommendation adopted by the Subsidiary Body on Implementation on 29 May 2024
CBD/SBI/REC/4/1 29 May 2024 https://www.cbd.int/doc/recommendations/sbi-04/sbi-04-rec-01-en.pdf

---

[7] Decision 15/4, annex, sect. C.

[8] Ibid., sect. C

4/1. Review of implementation: progress in the preparation of revised and updated national biodiversity strategies and action plans and the establishment of national targets in alignment with the Kunming-Montreal Global Biodiversity Framework

The Subsidiary Body on Implementation ….

1. Urges Parties to revise or update their national biodiversity strategies and action plans in accordance with Article 6 of the Convention on Biological Diversity,9 as requested in paragraph 6 of decision 15/6 of 19 December 2022, taking **section C** of the Kunming-Montreal Global Biodiversity Framework[10] into account;

Recommendation adopted by the Subsidiary Body on Implementation on 29 May 2024
https://www.cbd.int/doc/recommendations/sbi-04/sbi-04-rec-02-en.pdf

4/2. Mechanisms for planning, monitoring, reporting and review

The Subsidiary Body on Implementation ….

[Annex II Reporting of commitments by non-State actors[*]

26. Potential ways to address challenges and opportunities in achieving effective implementation of the commitment and its contribution to the Framework, including its **section C** and its targets and goals, and other decisions (e.g. Gender Plan of Action (2023–2030)).[11] (optional)

[9] United Nations, *Treaty Series*, vol. 1760, No. 30619.

[10] Decision 15/4, annex.

[*] [Reporting is voluntary and open to actors beyond national Governments, including indigenous peoples and local communities, the private sector and all relevant organizations and stakeholders.]

[11] Decision 15/11, annex.
